# MCAM Expression Facilitates Melanoma–Endothelial Interactions and Promotes Metastatic Disease Progression

**DOI:** 10.1111/exd.70059

**Published:** 2025-02-13

**Authors:** Andreas Dominik Braun, Miriam Mengoni, Thomas Tüting, Evelyn Gaffal

**Affiliations:** ^1^ Laboratory for Experimental Dermatology, Department of Dermatology University Hospital Magdeburg Magdeburg Germany; ^2^ Department of Dermatology, Allergy, and Venereology University of Lübeck Lübeck Germany

**Keywords:** cell adhesion, MCAM, melanoma, metastasis, migration

## Abstract

Invasive growth and metastatic dissemination represent the primary cause of death in cancer patients. In order to successfully detach from the primary tumour and establish metastases in distant tissues, cancer cells need to dynamically rewire their cell adhesion machinery. Here we revisit the potential association of MCAM, a member of the immunoglobulin superfamily that was initially identified as a melanoma antigen, with disease progression. Using immunohistochemical stainings and bioinformatic analyses of published datasets, we find abundant MCAM expression both in primary and metastatic human melanomas. In additional bioinformatic analyses, we show that MCAM is highly expressed in foetal melanocytes and subsequently downregulated during melanocyte maturation. Bioinformatic inference of cellular communication networks reveals that melanoma cells with high MCAM expression more actively engage in signalling crosstalk with endothelial cells. Experimental investigations demonstrate that disruption of MCAM in melanoma cells inhibits their migration on endothelial cell surfaces in vitro and decreases their ability to develop spontaneous lung metastases in vivo. Taken together, our results could not confirm the notion that MCAM expression represents a useful biomarker for disease progression but provide evidence that MCAM expression might represent part of a reactivated embryonal transcriptional program that facilitates melanoma–endothelial cell interactions during metastatic progression.

## 1 Introduction

Invasive growth and metastatic dissemination are the primary cause of death in patients with cancer [1]. In order to detach from the primary tumour and successfully invade distant tissues to form metastases, cancer cells need to rewire their cell adhesion machinery [2]. As a prototype of this process, cells from many cancer types have been shown to downregulate the adhesion molecule E‐cadherin and upregulate the related molecule N‐cadherin in a process termed epithelial to mesenchymal transition [3]. In addition to cadherins, also members from the integrin and immunoglobulin superfamily have been implicated in the migration of cancer cells [4].

As a prominent example of a cell adhesion molecule of the immunoglobulin superfamily, the melanoma cell adhesion molecule (MCAM) has been described to drive tumour progression in multiple cancer types [5]. Initially, MCAM was described as a tumour antigen expressed in primary melanomas but not benign melanocytic nevi [6]. Subsequent studies reported an increase of MCAM expression in melanoma cells during metastatic disease progression [7]. The specificity of MCAM in malignant lesions has been challenged by observations identifying abundant MCAM expression also in benign melanocytic nevi [8].

First experimental evidence for a tumour promoting role of MCAM was obtained through transgenic overexpression of MCAM in the melanoma cell line SB‐2, which resulted in an accelerated tumour growth of MCAM overexpressing cells after transplantation in mice [9]. Furthermore, MCAM has been described to mediate adhesion between melanoma and endothelial cells in vitro [10]. How MCAM promotes tumour invasion and metastatic spread is currently not fully understood.

In our current work, we combine immunohistopathologic analyses of 69 advanced human primary melanomas and 42 melanoma metastases, bioinformatic analyses of published transcriptome datasets, and experimental studies in a mouse melanoma model to address the function of MCAM in melanoma progression.

## 2 Materials and Methods

### 2.1 Cell Culture

HCmel12 mouse melanoma cells were derived from the Hgf‐Cdk4 mouse melanoma model as previously described [11]. Cells were cultivated in the Roswell Park Memorial Institute (RPMI) 1640 medium (Life Technologies) supplemented with 10% foetal calf serum (Biochrome), 2 mM L‐glutamine (Life Technologies), 10 mM non‐essential amino acids (Life Technologies), 1 mM 4‐(2‐hydroxyethyl)‐1‐piperazine‐1‐ethane‐sulfonic acid (HEPES, Life Technologies), 20 μM β‐mercaptoethanol (Sigma), 100 IU/mL penicillin and 100 μg/mL streptomycin (Invitrogen). Cultivation was performed in a humidified incubator at 5% CO_2_. Cells were screened for contamination with mycoplasma with no detection of mycoplasma.

### 2.2 Patient Material

A total of 69 primary melanomas and 42 melanoma metastases were obtained during routine patient care at the Department for Dermatology of the University Hospital Magdeburg. Sample processing and haematoxylin and eosin staining were performed using standard histopathologic procedures. Immunohistochemistry was performed using an automated Ventana BenchMark with anti‐MCAM (1:200, Epitomics, catalogue #AC‐0052), anti‐SOX10 (Master diagnóstica, catalogue #MAD‐000656QD) and anti‐CD31 antibodies (Cell Marque, catalogue #131M‐98). Usage of the routinely acquired material for research was approved by the ethics committee of the Otto‐von‐Guericke University Magdeburg (approval number 162/20), and informed consent was obtained from patients. All experiments were performed in accordance with local ethical and legal regulations.

Digital whole slide images of MCAM‐stained sections were classified into four stain grades by trained dermatopathologists. Stain grade 3 indicates a ubiquitous strong staining in a similar intensity to the adjacent endothelial cells. Stain grade 2 was assigned to samples showing strong staining, but also contained areas with weaker or no staining. Stain grade 1 was assigned to samples with detectable staining without showing strong expression levels when compared to adjacent endothelial cells. Stain grade 0 was assigned to samples showing almost no MCAM staining. Only MCAM staining on tumour cells was considered for the classification.

### 2.3 CRISPR/Cas9 Knockout of MCAM

To create MCAM knockout cells, 5 × 10^5^ HCmel12 melanoma cells were seeded into a 12‐well plate and transfected with 1.6 μg MCAM sgRNA plasmid and 0.4 μg pRP‐TagGFP2 plasmid using the FuGene HD Transfection System (Promega) according to manufacturer's instructions. Control cells were transfected with an empty CRISPR vector without containing sgRNA. As the sgRNA backbone, the plasmid px330 (addgene Plasmid #42230) was used. Fluorescently labelled single cells were sorted using a FACSAria III cell sorter (BD) into 96‐well plates. Genomic DNA from monoclones was isolated with the NucleoSpin Tissue kit (Macherey‐Nagel) according to manufacturer's protocol. The MCAM knockout target region was amplified via PCR and subsequently sequenced on a MiSeq Sequencer (Illumina) in single‐end mode for 300 cycles. Successful frameshift mutations were identified using CRIS.py [12].

### 2.4 Immunoblot

Proteins from HCmel12 cells were isolated using the mammalian protein extraction reagent (Fermentas) supplemented with protease inhibitors (Thermo Scientific). Protein concentrations were quantified using the Pierce BCA protein assay kit (Thermo Scientific) and measurement on a microplate reader at 562 nm (Tecan Group). Subsequently, 10 μg of protein was mixed with Roti Load loading buffer (Roth) and separated by SDS‐PAGE. Proteins were transferred to a PVDF membrane with 0.45 μm pore size (GE Healthcare) by wet blotting. Membranes were blocked with 5% skim milk for 1 h and stained using the anti‐MCAM antibody (1:2000, Thermo Scientific, catalogue #14‐1469‐82, RRID AB_1210462) overnight at 4°C for the primary antibody, and the anti‐mouse IgG HRP (1:3000, Cell Signalling, catalogue #7076) for 1 h at room temperature. As loading control, a HRP‐conjugated anti‐b‐actin antibody was used (1:5000, Santa Cruz, catalogue #sc‐47 778 HRP). Detection was performed using the SignalFire ECL reagent (Cell Signalling) and the chemiluminescence acquired using an Octuplus QPLEX imager (NH DyeAgnostics).

### 2.5 In Vitro Melanoma‐Endothelial Co‐Culture Migration Assay

Mouse endothelial cells (bEND) were plated at a density of 10^5^ cells in a μ‐Slide eight‐well chambered coverslip (Ibidi) and incubated for 16 h at 37°C and 5% CO_2_. Next, 10^4^ TagGFP2‐labelled HCmel12 CRISPR control or a mixture of three HCmel12 MCAM knockout clones in equal proportion were carefully seeded on top of the attached endothelial cells. Migration was followed using a fully automated Leica TCS SP8 confocal microscope equipped with a climate chamber (37°C, 5% CO_2_ with humidity). Images were acquired every 5 min for 12 h using a 10× objective. For each well, three representative viewing fields were captured using the track‐and‐mark feature. Migration distance and velocity of individual cells were quantified from image stacks using ImageJ with the TrackMate plugin [13]. Cells with a track duration < 30 min and track length < 50 mm were removed from further analysis.

### 2.6 Transwell Migration Assay

Mouse endothelial cells (bEND) were plated at a density of 5 × 10^4^ cells either on the upper side of a FluoroBlok transwell membrane (Corning) or in the lower compartment of a boyden chamber separated by a FlouroBlok transwell membrane and incubated for 2 h at 37°C and 5% CO_2_. Next, 2 × 10^4^ TagGFP2‐labelled HCmel12 CRISPR control or a mixture of three HCmel12 MCAM knockout clones in equal proportion were seeded on top of the transwell and incubated for 16 h at 37°C and 5% CO_2_. Migrated cells were imaged on the lower side of the transwell membrane using an inverted fluorescence microscope (AxioVert A1, Zeiss) equipped with a mercury lamp (HXP120, Zeiss). Three representative viewing fields at 10× magnification were captured using an Axiocam microscope camera (Zeiss). Migrated cells were quantified from images automatically using PyImageJ. The workflow consisted of background subtraction using the function ‘Subtract Background …’ with the parameter ‘rolling = 50’, setting of a raw threshold using the function ‘setRawThreshold’ with the parameters ‘(10, 255)’, conversion of the image format to a mask using the function ‘Convert to Mask’ and finally the counting of cells using the function ‘Analyse Particles …’ with the parameters ‘size=5‐Infinity clear overlay’.

### 2.7 Tumour Transplantation Experiments

C57BL/6J mice were acquired from Janvier or were taken from own breeding. In all, 2 × 10^5^ HCmel12 CRISPR control cells or a mixture of three HCmel12 MCAM knockout clones in equal proportion were transplanted intracutaneously into the right flank using a 30G needle (BD). Tumour growth monitoring was performed three times per week using a vernier calliper and recorded as mean diameter. Mice were euthanized when tumours exceeded 20 mm in diameter or when signs of illness in accordance with local ethical regulations were observed. Macroscopic counting of lung metastases was conducted by inspection. Mice were age and sex matched, and randomly assigned to experimental groups at the start of each experiment. All experiments were conducted using groups of six mice and repeated independently at least twice. Experiments were performed in accordance with local ethical and legal regulations with the approval of the responsible authorities (Landesverwaltungsamt Saxony‐Anhalt, Germany, approval number: 42502‐2‐1393 Uni MD).

### 2.8 Bioinformatic Analysis of Published Datasets

TCGA bulk RNA sequencing data for cutaneous melanoma samples were downloaded from cBioPortal as rsem normalised count matrix [14, 15]. For the survival analysis, MCAM expression in samples was binarised using the mean as threshold. Clinical data including overall survival was also obtained from cBioPortal. The raw count matrix of single‐cell RNA sequencing data from human melanocytes was obtained from GEO (accession number GSE151091) [16] and loaded into Scanpy [17]. Data were preprocessed by filtering cells with < 50.000 unique reds, < 500 genes and a fraction of ERCC spike reads > 20%. Genes detected in < 3 cells were removed from further analysis. Doublets detection was performed using scrublet [18], and detected doublets removed. Reads were normalised using the size factor as calculated using scran [19]. Mouse single–cell RNA sequencing data from the NRAS/Ink4a model were obtained from the Marine group as published (https://drive.google.com/drive/folders/1poq4Lo5AxVp0WpG1EMgIjIeDR4q98zcA) [20]. The cell‐type annotation was used as published, with subsequent annotation of malignant cells as MCMA high for cells with MCAM expression greater than the cohort median + SD. The interaction analysis was performed using CellChatv2 [21] as outlined in the documentation using standard parameters. Bulk RNA sequencing data from mouse melanocytes was obtained from GEO (accession number GSE140193) [22]. For the heat map, genes with a spearman correlation coefficient > 0.5 with age in the human melanocyte dataset were shown.

## 3 Results

### 3.1 MCAM Is Highly Expressed in Both Primary and Metastatic Melanomas and Does Not Significantly Increase Between Matched Primary Tumours and Metastases

**FIGURE 1:**
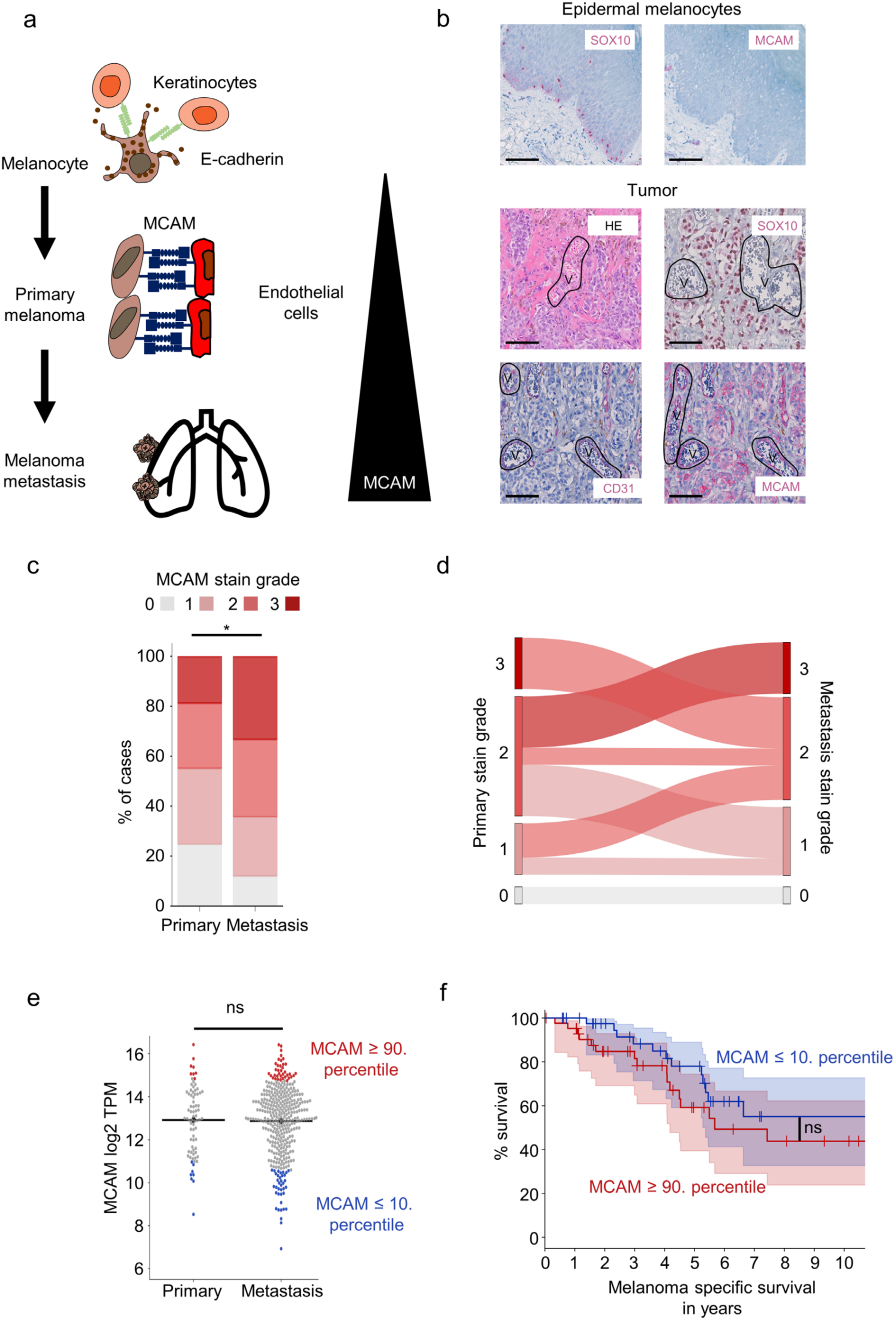
MCAM is highly expressed in both primary and metastatic melanomas and does not significantly increase between matched primary tumours and metastases. (a) Conceptual outline and overview of the current work showing the working hypothesis of MCAM as a mediator of cell–cell interactions between melanoma and endothelial cells promoting angiotropic growth and metastatic dissemination. (b) Representative immunohistochemistry stainings of vessels, intraepidermal melanocytes and melanomas. Scale bar indicates 100 μm. (c) Distribution of MCAM stain grades in 69 primary melanomas and 42 melanoma metastases. * = *p* < 0.05, Mann–Whitney‐*U* test. (d) Comparison of MCAM stain grades from matched primary and metastatic melanomas in 14 patients. (e) mRNA expression of MCAM in primary and metastatic melanoma samples from the TCGA SKCM cohort. The bars indicate the mean. For samples with MCAM expression ≥ 90, percentiles are marked in red, and for samples with MCAM expression ≤ 10, percentiles are marked in blue. ns = not significant, unpaired two‐sided *t*‐test. (f) Kaplan–Meier curves of melanoma‐specific survival for TCGA SKCM cohort stratified by MCAM expression. ns = not significant, log‐rank test.

In initial studies, we reassessed the value of MCAM as a potential marker for melanoma progression (Figure 1a). We stained sections of advanced primary melanomas with a vertical tumour thickness ≥ 1 mm that were derived from 69 patients (Table 1) for MCAM, the melanoma marker So × 10 and the endothelial marker CD31 using immunohistochemistry. As previously reported, MCAM was not expressed on epidermal melanocytes but on many melanoma cells with high intra‐ and intertumoral heterogeneity (Figure 1b). In addition, endothelial cells constitutively expressed MCAM (Figure 1b). We quantified the expression of MCAM in tumour cells, taking into account both the stain area and intensity, and observed MCAM expression in most melanomas (Figure S1a,b). In contrast to previous reports [7, 8], we did not observe a significant correlation between MCAM staining and vertical tumour thickness in our cohort (Figure S1b). We also stained sections of melanoma skin and lymph node metastases derived from 42 patients and found MCAM expression to be slightly increased compared to primary melanomas (Figure 1c). An analysis of the subset of matched primary and metastatic melanomas derived from 14 patients did not show a clear increase of MCAM staining during disease progression (Figure 1d).

**TABLE 1:** Characteristics of the primary melanoma cohort.

Characteristic	Total (*n* = 69)
Age*	71 (59–78)
Sex: male	43 (62.3%)
Sex: female	26 (37.7%)
Vertical tumour thickness*	4.3 (2.8–7.0)
Location: head/neck	14 (20.3%)
Location: trunk	24 (34.8%)
Location: upper extremity	10 (14.5%)
Location: lower extremity	21 (30.4%)
Type: SSM	19 (27.5%)
Type: nodular	30 (43.5%)
Type: ALM	12 (17.4%)
Type: LMM	1 (1.4%)
Type: other	7 (10.1%)
Sentinel positive	18 (26.1%)
Sentinel negative	38 (55.1%)
Sentinel not performed	13 (18.8%)
BRAF wild type	16 (23.2%)
BRAF mutated	13 (18.8%)
BRAF not tested	40 (58.0%)
Stage: IA	1 (1.4%)
Stage: IB	6 (8.7%)
Stage: IIA	8 (11.6%)
Stage: IIB	9 (13.0%)
Stage: IIC	12 (17.4%)
Stage: IIIA	1 (1.4%)
Stage: IIIB	3 (4.3%)
Stage: IIIC	24 (34.8%)
Stage: IIID	1 (1.4%)
Stage: IV	4 (5.8%)

*Note:* Data are shown as absolute number of patients with percentages in parentheses, with the exception for marked values (*), where median values are shown, and interquartile ranges are given in parentheses.

Next, we evaluated the expression of MCAM in bulk RNA sequencing data of the ‘Cancer Genome Atlas’ project [14]. This bioinformatic analysis revealed expression of MCAM mRNA in both primary and metastatic melanomas, with no significant differences of MCAM mRNA expression levels between primary and metastatic melanomas (Figure 1e). Moreover, we stratified patients into cohorts with MCAM mRNA expression above the 90th percentile and below the 10th percentile. Patients from these cohorts showed a trend towards improved melanoma‐specific survival for patients with low MCAM expression compared to patients with high MCAM expression without reaching statistical significance (Figure 1f). In summary, these results show that MCAM is abundantly expressed in both primary and metastatic melanomas.

### 3.2 MCAM Is Expressed on Foetal Melanocyte Precursors and Downregulated During Maturation

**FIGURE 2:**
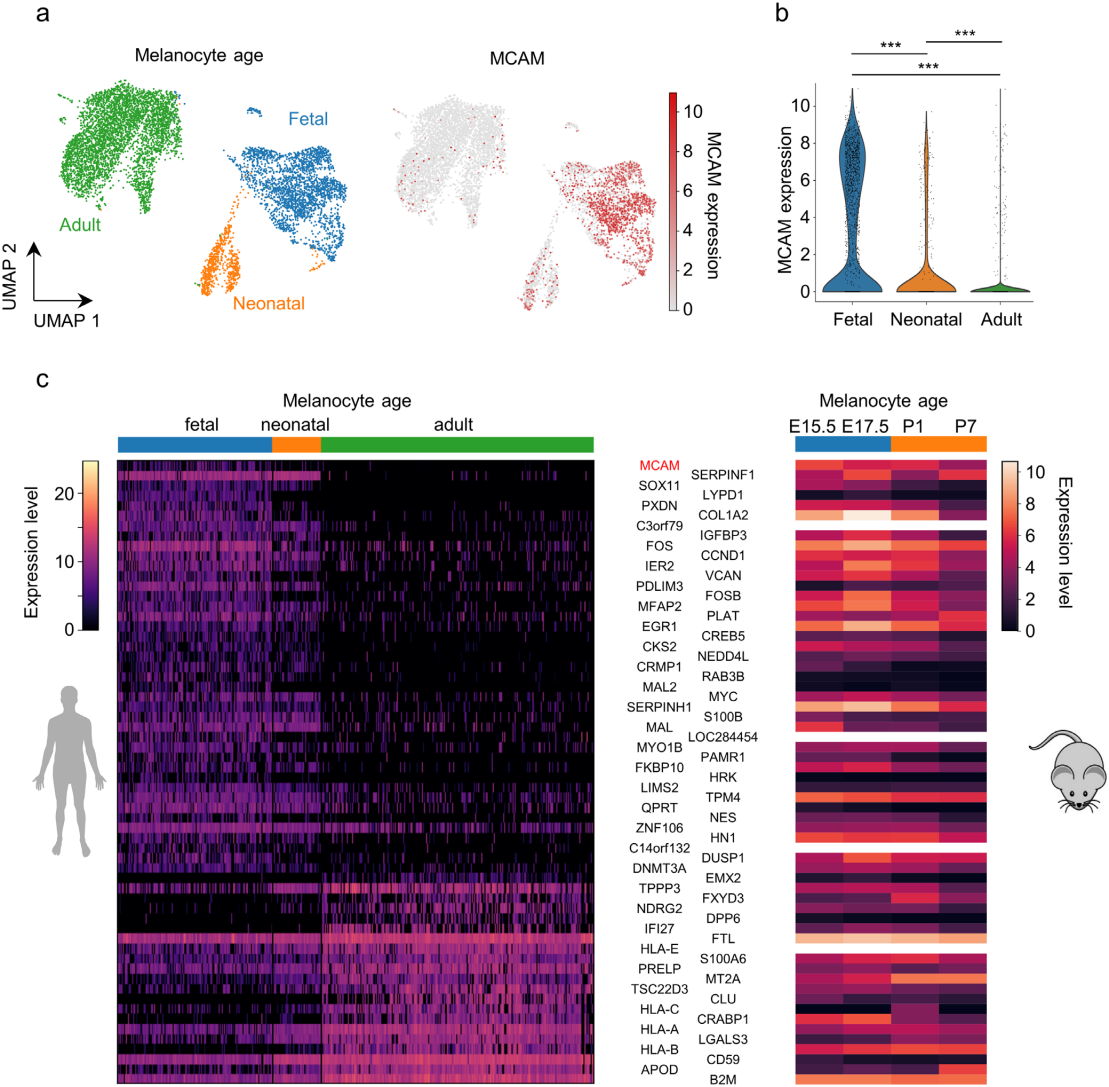
MCAM is expressed on foetal melanocyte precursors and downregulated during maturation. (a) Dimensionality reduction and visualisation of single‐cell transcriptomes from melanocytes from different developmental stages using UMAP. Data were obtained from the GEO (GSE151091). (b) MCAM expression in single cells grouped by developmental stage. *** = *p* < 0.001, Kruskal–Wallis test with post hoc Mann–Whitney‐*U* test and Bonferroni correction. (c) Heat map of genes correlated developmental stage in human melanocytes. Shown are all genes with a Spearman correlation coefficient > 0.5. The same genes are also shown for mouse melanocytes obtained at different ages (GSE140193). E15.5/E17.5 = embryonic day 15.5/17.5, P1/P7 = 1/7 days post birth.

Metastatic dissemination of melanoma cells has been associated with the ability to closely interact with endothelial cells and to acquire dedifferentiated and stem‐like cell states [11, 20]. In this process, melanoma cells are thought to reactivate the migratory abilities of their neural crest precursors. We therefore hypothesised that the upregulation of MCAM on melanoma cells might reflect an embryonal transcriptional program. We addressed this hypothesis in bioinformatic analyses of a scRNA sequencing dataset obtained from human melanocytes at different developmental stages [16]. Whereas MCAM mRNA expression was strongly expressed in many foetal melanocytes, we detected only few neonatal and hardly any adult melanocytes with high MCAM mRNA levels (Figure 2a,b). Next, we calculated spearman rank correlation coefficients for each gene across melanocyte developmental stages. Interestingly, MCAM was the only cell adhesion molecule that showed a significant inverse correlation with melanocyte maturation (Figure 2c). In another bulk RNA sequencing dataset from mouse melanocytes sampled at different developmental stages [22], MCAM expression was also downregulated during melanocyte maturation, indicating an evolutionary conserved role of MCAM during melanocyte maturation (Figure 2c).

### 3.3 Bioinformatic Analyses Support a Functional Role for MCAM in Melanoma Cell—Endothelial Cell Interactions

**FIGURE 3:**
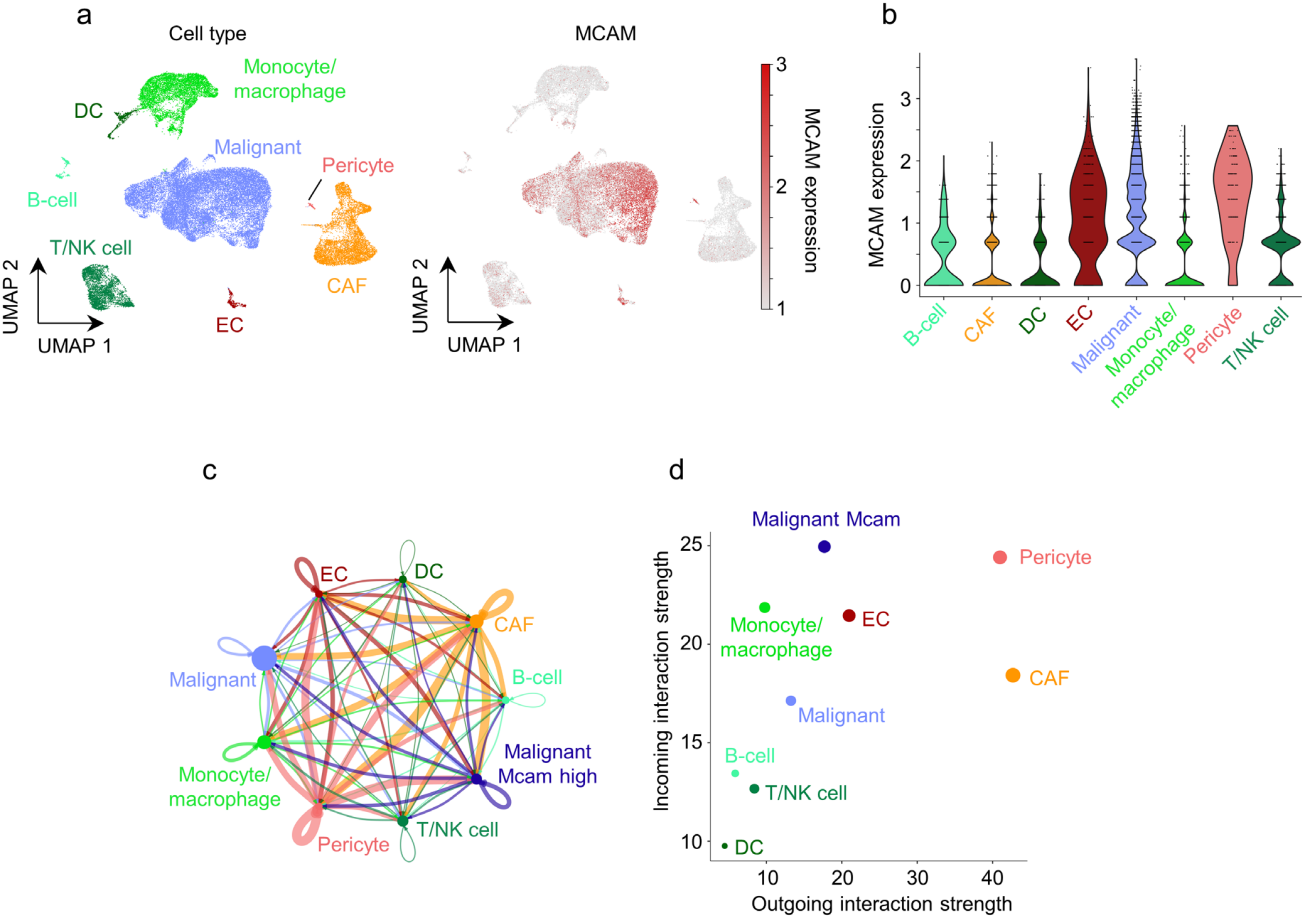
Bioinformatic analyses suggest a functional role for MCAM in melanoma cell–endothelial cell interactions. (a) Dimensionality reduction and visualisation of single‐cell transcriptomes from melanocytes from the Nras/Ink4a mouse melanoma model using UMAP. Data were obtained from the Marine group as published [20]. (b) MCAM expression in single cells grouped by cell type. (c) Computationally inferred interaction network between cell types performed with CellChatv2. The width of the lines indicates the signalling strength and the diameter of the dots indicates the group sizes. (d) Incoming and outgoing signal strength from computationally inferred interactions for each cell type. EC: endothelial cell, DC: dendritic cell, CAF: cancer‐associated fibroblast.

During embryonal development, neural crest cells have been demonstrated to interact with endothelial cells [23]. Due to the high expression of MCAM on both melanoma and endothelial cells, we hypothesised that MCAM might also promote the interaction of melanoma and endothelial cells. In order to test this hypothesis, we analysed a scRNA sequencing dataset of mouse melanomas from the Nras/Ink4a model [20]. As expected, we detected high expression levels of MCAM in melanoma cells, endothelial cells and pericytes (Figure 3a,b). We next used CellChat to computationally infer cellular interaction networks [21]. In this analysis, we were able to detect a tight interaction network between melanoma cells, endothelial cells, pericytes and cancer‐associated fibroblasts (Figure 3c). Interestingly, this interaction was stronger in melanoma cells with high MCAM expression compared to melanoma cells with low MCAM expression (Figure 3d), suggesting that MCAM promotes the interaction of melanoma cells to endothelial cells and pericytes.

### 3.4 MCAM‐Deficient HCmel12 Mouse Melanoma Cells Seeded Onto Endothelial Cell Monolayers In Vitro Show Reduced Motility

**FIGURE 4:**
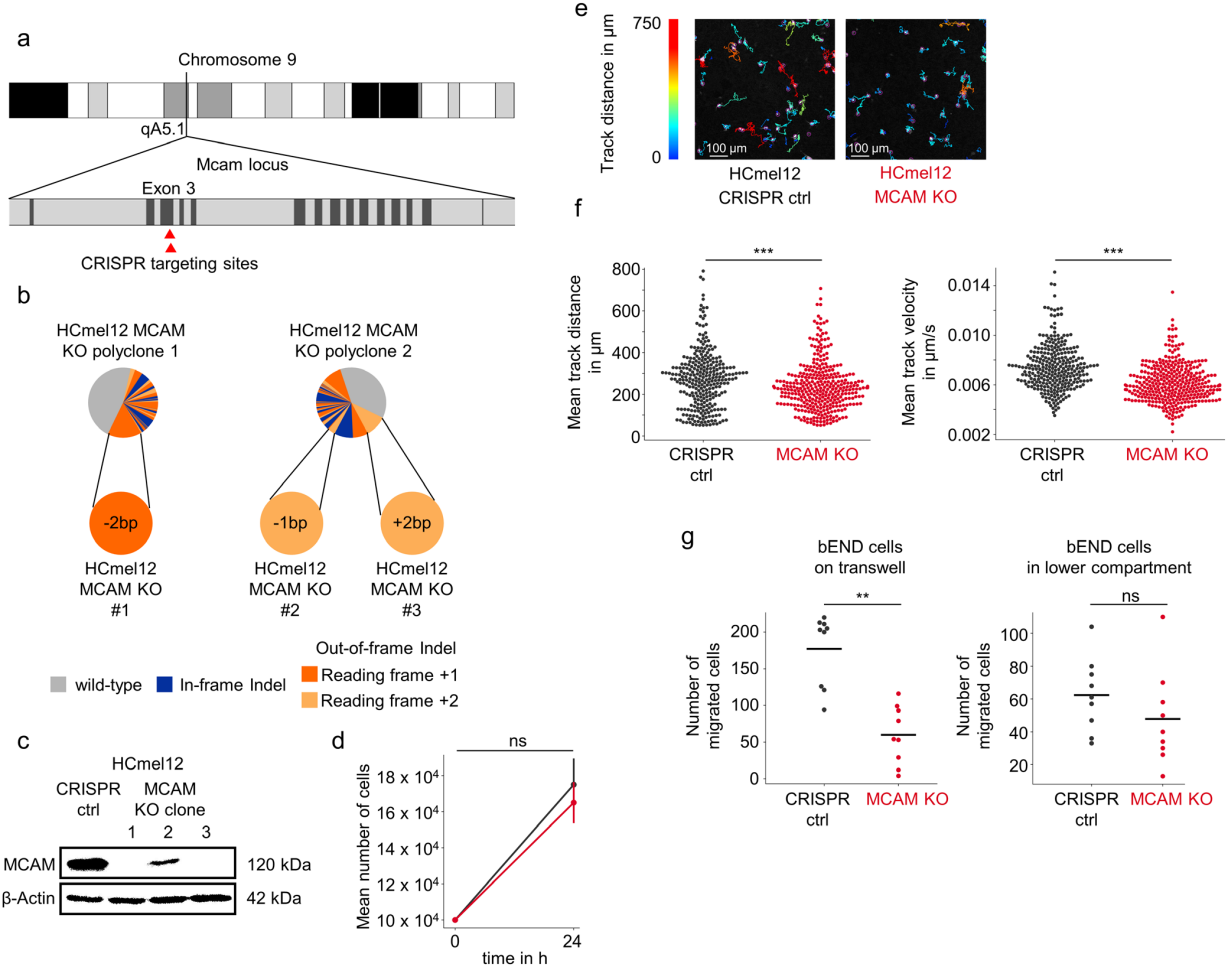
MCAM‐deficient HCmel12 mouse melanoma cells show reduced migratory activity on endothelial cell monolayers. (a) Chromosomal position of Mcam gene locus in mice. Red triangles indicate the guide RNA targeting sites. (b) Next generation sequencing of HCmel12 MCAM CRISPR knockout poly‐ and monoclones, showing homozygous out‐of‐frame mutations for the three monoclones used in further experiments. (c) Immunoblot of three HCmel12 MCAM CRISPR knockout monoclones. (d) Cell counting assay of HCmel12 CRISPR control and MCAM knockout cells. Shown are mean values from three biological replicates. Error bars indicate the standard deviation. ns = not significant, Student's *t*‐test. (e) Representative images of in vitro melanoma–endothelial co‐culture migration assay. For the MCAM knockout cells, a mixture of the three HCmel12 MCAM knockout clones were used in equal proportion. Images are shown at 10× magnification. Colours indicate individual tracks as constructed by TrackMate. (f) Mean track distance and velocity for individual melanoma cells. *** = *p* < 0.001, Mann–Whitney *U* test. (g) Migrated cells through transwell for HCmel12 CRISPR control and MCAM knockout cells. For the MCAM knockout cells, a mixture of the three HCmel12 MCAM knockout clones were used in equal proportion. bEND endothelial cells were either seeded on the transwell (left) or in the lower compartment (right) 2 h prior to the seeding of melanoma cells on the transwell. ** = *p* < 0.005, ns = not significant, Mann–Whitney *U* test.

To experimentally confirm the hypothesis that MCAM promotes the interaction of melanoma and endothelial cells, we generated MCAM knockout mouse melanoma cells (Figure 4a). For this, we used the cell line HCmel12, which has been shown to readily migrate on endothelial cells in vitro [11]. Disruption of the MCAM gene was confirmed via next generation sequencing, and MCAM knockout validated on protein level via immunoblot (Figure 4b,c). No significant differences in the proliferation rates were observed between HCmel12 MCAM knockout and HCmel12 CRISPR control cells (Figure 4d). We then followed migration of HCmel12 MCAM knockout and HCmel12 CRISPR control cells on endothelial cell monolayers in vitro using time‐lapse video microscopy (Figure 4e). In agreement with our hypothesis, we observed a significant reduction in migration distance and velocity of HCmel12 MCAM knockout cells compared to HCmel12 CRISPR control cells (Figure 4f). Next, we performed a transwell migration assay seeding bEND endothelial cells either in the top compartment on the transwell or in the lower compartment. Whereas a significantly higher number of HCMel12 CRISPR control cells migrated through the transwells with bEND cells seeded alongside on the transwell compared to HCmel12 MCAM knockout cells, fewer cells migrated through the transwell with bEND cells seeded in the lower compartment without statistically significant differences between HCmel12 CRISPR control and MCAM knockout cells (Figure 4g). These results indicate that a direct interaction between endothelial cells and melanoma cells is required for MCAM to promote angiotropic migration.

### 3.5 MCAM‐Deficient HCmel12 Mouse Melanoma Cells Transplanted Into the Skin In Vivo Show Reduced Numbers of Spontaneous Lung Metastasis

**FIGURE 5:**
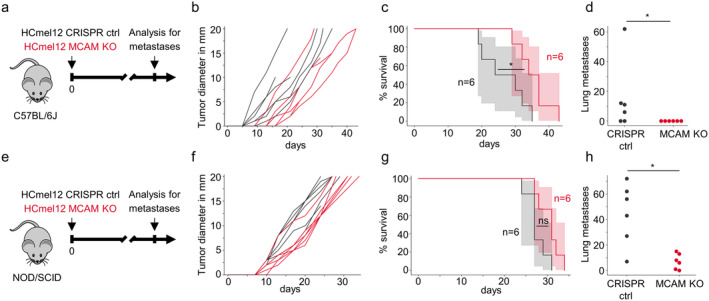
MCAM‐deficient HCmel12 mouse melanoma cells transplanted into the skin in vivo show reduced numbers of spontaneous lung metastasis. (a) Experimental protocol for the analysis of tumour growth and spontaneous lung metastasis of HCmel12 CRISPR control cells and a mixture of the three HCmel12 MCAM knockout clones in equal proportion. Lung metastases were quantified by visual inspection at the time of death. The experiment was performed twice. (b, c) Tumour growth (b) and Kaplan–Meier survival curves (c) of C57BL/6 mice bearing established tumours. * = *p* < 0.05, log‐rank test. (d) Number of spontaneous macroscopic lung metastases in C57BL/6 mice. * = *p* < 0.05, Mann–Whitney *U* test. (e) Experimental protocol for the analysis of tumour growth and spontaneous lung metastasis of HCmel12 CRISPR control and a mixture of the three HCmel12 MCAM knockout clones in equal proportion in NOD/SCID mice. Lung metastases were quantified by visual inspection at the time of death. The experiment was performed twice. (f, g) Tumour growth (f) and Kaplan–Meier survival curves (g) of NOD/SCID mice bearing established tumours. ns = not significant, log‐rank test. (h) Number of spontaneous macroscopic lung metastases in NOD/SCID mice. * = *p* < 0.05, Mann–Whitney *U* test.

The angiotropic growth of melanoma cells has been reported to promote melanoma metastatic spread [11]. Because the knockout of MCAM disrupted the migration of melanoma cells on endothelial cells, we hypothesised that MCAM knockout might also reduce metastatic dissemination of melanoma cells. To experimentally test this hypothesis, we intradermally transplanted HCmel12 CRISPR control and HCmel12 MCAM knockout cells in syngeneic BL6 mice (Figure 5a). Mice transplanted with MCAM knockout cells showed a longer survival (Figure 5b,c) compared to HCmel12 CRISPR control cells. Whereas mice bearing HCmel12 CRISPR control melanomas frequently developed macroscopically visible lung metastases, no lung metastases were observed in mice transplanted with HCmel12 MCAM knockout cells (Figure 5d). To further dissect the role of MCAM on the tumour‐cell intrinsic metastatic potential or the impact of MCAM on immune evasion during metastatic dissemination, we intradermally transplanted HCmel12 CRISPR control and HCmel12 MCAM knockout cells in NOD/SCID mice (Figure 5e). HCmel12 MCAM knockout cells developed significantly fewer lung metastases compared to their MCAM proficient HCmel12 CRISPR control counterparts in NOD/SCID mice (Figure 5f–h). These results demonstrate that MCAM promotes metastatic dissemination of melanoma cells.

## 4 Discussion

MCAM has previously been described as a marker of melanoma disease progression and metastasis [7, 8]. Here, we profiled MCAM protein expression using immunohistochemistry and MCAM mRNA expression using bioinformatic analyses of published transcriptome datasets. In our work, we observed a frequent expression of MCAM in most primary and metastatic melanomas. Due to the abundant expression of MCAM already in many primary lesions, our data do not support the role of MCAM mRNA or protein expression levels as a biomarker for melanoma progression. Furthermore, the analysed TCGA bulk RNA sequencing data provide only an integral of the MCAM expression over all cell types and melanoma cell phenotypes without the ability to identify the abundance of the MCAM high stem–like melanoma cells, additionally limiting the applicability of MCAM mRNA levels measured in bulk RNA sequencing as a biomarker. However, the wide expression of MCAM on melanoma cells might make it a candidate for identifying circulating melanoma cells as a marker of tumour burden and disease recurrence as previously described [24, 25].

In our bioinformatic analyses, we find abundant expression of MCAM in developmental melanocyte precursors, suggesting that the expression of MCAM in melanoma cells might reflect the reacquisition of stem‐like phenotypes of their embryonal precursors. Interestingly, recent reports also identified MCAM expression as a marker of migrating developmental neural crest cell phenotypes [26], further supporting a role of MCAM in melanocyte development and migration. Bioinformatic inference of cellular communication networks revealed that melanoma cells with high MCAM expression more actively engage in signalling crosstalk with endothelial cells. The close interaction of melanoma cells with vessels, a process known as angiotropism, has previously been identified as a driver of melanoma metastasis [11, 27, 28]. In addition to securing the supply of oxygen and nutrients, melanoma–endothelial interactions have been demonstrated to promote stem–like, migratory melanoma cell phenotypes [20, 29].

Our observation that a CRISPR‐Cas9–mediated disruption of the MCAM gene in melanoma cells impairs their ability to migrate on endothelial cell surfaces in vitro and decreases their ability to develop spontaneous lung metastases in vivo functionally support the notion that MCAM mediates melanoma–endothelial interactions and thereby facilitates metastatic progression. MCAM on melanoma cells could interact with molecules such as Laminin‐411 that are highly expressed in vessel walls and have been described as ligands for MCAM [30]. MCAM has also been described as a co‐receptor for several growth factor receptors such as VEGFR2 or PDGFR‐β or inflammatory mediators such as S100A8/A9, and may thereby promote cell growth and simulate tumour vascularisation [31, 32, 33, 34, 35]. In addition to MCAM‐mediated interactions, also other signalling mechanisms have been described to mediate melanoma–endothelial crosstalk such as Dll4‐Notch3 signalling [20]. The exact molecular mechanisms how MCAM facilitates melanoma–endothelial interactions remain to be determined in future work.

## Author Contributions

A.D.B. and M.M. performed experiments and analysed data. M.M. and A.D.B. collected clinical data. A.D.B. performed bioinformatics analyses. A.D.B. and T.T. evaluated histopathological sections. A.D.B., T.T. and E.G. designed experiments. A.D.B., M.M., T.T. and E.G. contributed intellectual input and helped to interpret data. E.G. supervised the research project. A.D.B., M.M., T.T. and E.G. wrote and reviewed the manuscript.

## Ethics Statement

All animal experiments were conducted in accordance with the ARRIVE guidelines and in compliance with federal and international guidelines with the approval of the responsible authorities (Landesverwaltungsamt Saxony‐Anhalt, Germany, approval number: 42502‐2‐1393 Uni MD). The use of the routinely acquired patient‐derived melanoma material for research was approved by the ethics committee of the Otto‐von‐Guericke University Magdeburg (approval number 162/20).

## Conflicts of Interest

The authors declare no conflicts of interest.

## Supporting information


**Figure S1.** MCAM stain grade in primary melanomas does not correlate with vertical tumour thickness (a) Representative immunohistochemical stains of human primary melanomas and melanoma metastases grouped by MCAM stain grades. Scale bar indicates 500 μm. (b) Comparison of MCAM stain grades with tumour thickness of primary melanomas. The size of the dots indicates the absolute number of samples and the colour indicates the relative proportion per thickness group. The spearman correlation coefficient is given above.

## Data Availability

The data that support the findings of this study are available from the corresponding author upon reasonable request.
