# Supplementary figures and images for: MCAM Expression Facilitates Melanoma–Endothelial Interactions and Promotes Metastatic Disease Progression
Source: Exp Dermatol. 2025 Feb 13;34(2):e70059. doi: 10.1111/exd.70059 (PMC11822558; doi:10.1111/exd.70059)

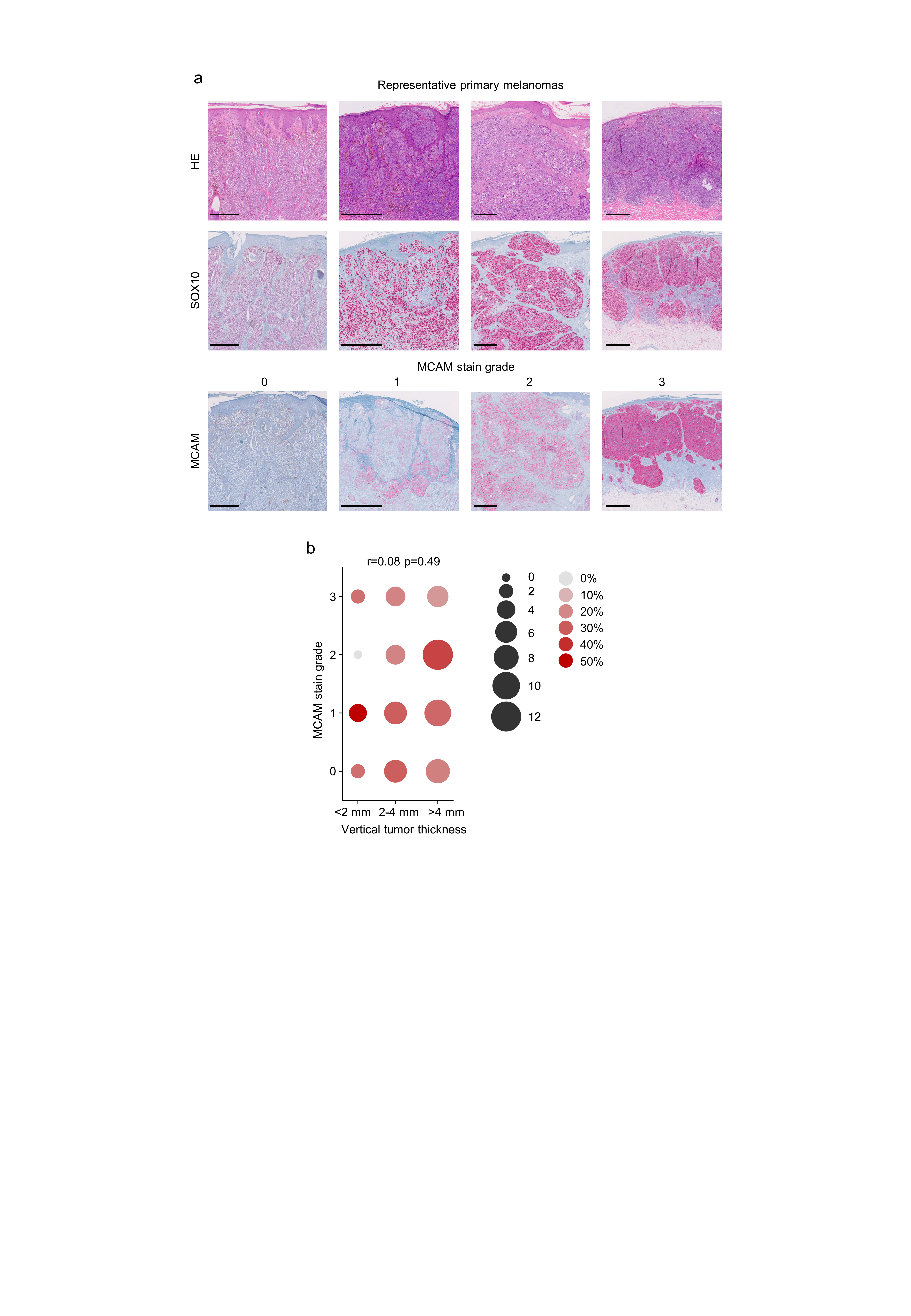

Supplement: Supplementary file 1 — Figure S1. MCAM stain grade in primary melanomas does not correlate with vertical tumour thickness (a) Representative immunohistochemical stains of human primary melanomas and melanoma metastases grouped by MCAM stain grades. Scale bar indicates 500 μm. (b) Comparison of MCAM stain grades with tumour thickness of primary melanomas. The size of the dots indicates the absolute number of samples and the colour indicates the relative proportion per thickness group. The spearman correlation coefficient is given above. [file EXD-34-e70059-s001.tif]
